# Assessing the Feasibility of Augmenting Fall Detection Systems by Relying on UWB-Based Position Tracking and a Home Robot

**DOI:** 10.3390/s20185361

**Published:** 2020-09-18

**Authors:** Maurizio Capra, Stefano Sapienza, Paolo Motto Ros, Alessio Serrani, Maurizio Martina, Alessandro Puiatti, Paolo Bonato, Danilo Demarchi

**Affiliations:** 1 Department of Electrical, Electronics and Telecommunication Engineering, Politecnico di Torino, 10129 Torino TO, Italy; paolo.mottoros@polito.it (P.M.R.); alessio.serrani@polito.it (A.S.); maurizio.martina@polito.it (M.M.); Danilo.demarchi@polito.it (D.D.); 2 Department of Physical Medicine and Rehabilitation, Harvard Medical School, Boston, MA 02129, USA; pbonato@mgh.harvard.edu; 3 Institute for Information Systems and Networking, University of Applied Sciences and Arts of Southern Switzerland, 6928 Manno, Switzerland; alessandro.puiatti@supsi.ch

**Keywords:** indoor positioning, localization, machine learning, tele-presence robot, ultra wide band, wireless sensor network

## Abstract

Falls in the home environment are a primary cause of injury in older adults. According to the U.S. Centers for Disease Control and Prevention, every year, one in four adults 65 years of age and older reports experiencing a fall. A variety of different technologies have been proposed to detect fall events. However, the need to detect all fall instances (i.e., to avoid false negatives) has led to the development of systems marked by high sensitivity and hence a significant number of false alarms. The occurrence of false alarms causes frequent and unnecessary calls to emergency response centers, which are critical resources that should be utilized only when necessary. Besides, false alarms decrease the level of confidence of end-users in the fall detection system with a negative impact on their compliance with using the system (e.g., wearing the sensor enabling the detection of fall events). Herein, we present a novel approach aimed to augment traditional fall detection systems that rely on wearable sensors and fall detection algorithms. The proposed approach utilizes a UWB-based tracking system and a home robot. When the fall detection system generates an alarm, the alarm is relayed to a base station that utilizes a UWB-based tracking system to identify where the older adult and the robot are so as to enable navigating the environment using the robot and reaching the older adult to check if he/she experienced a fall. This approach prevents unnecessary calls to emergency response centers while enabling a tele-presence using the robot when appropriate. In this paper, we report the results of a novel fall detection algorithm, the characteristics of the alarm notification system, and the accuracy of the UWB-based tracking system that we implemented. The fall detection algorithm displayed a sensitivity of 99.0% and a specificity of 97.8%. The alarm notification system relayed all simulated alarm notification instances with a maximum delay of 106 ms. The UWB-based tracking system was found to be suitable to locate radio tags both in line-of-sight and in no-line-of-sight conditions. This result was obtained by using a machine learning-based algorithm that we developed to detect and compensate for the multipath effect in no-line-of-sight conditions. When using this algorithm, the error affecting the estimated position of the radio tags was smaller than 0.2 m, which is satisfactory for the application at hand.

## 1. Introduction

The global population aged 65 and older is rapidly growing due to the increase in life expectancy and the decline in fertility rate. By 2050, older adults will represent on average 16% of the world population, and 25% of the population in Europe and North America. Falls are a major cause of morbidity and mortality in older adults [1]. For this reason, engineers have developed wearable systems to detect fall events, trigger alarms, and deploy prompt interventions [2,3,4,5]. Fall detection systems typically rely on a wearable sensor with an embedded Inertial Measurement Unit (IMU) and on machine learning algorithms designed to detect fall events based on the IMU data. Because instances when a fall event occurs and the system does not detect the event (i.e., false negatives) cannot be tolerated, fall detection systems are designed to operate with high sensitivity. Unfortunately, the high sensitivity of the system is likely to be associated with generating a considerable number of false alarms. Bagalà et al. [6] highlighted this problem by testing fall detection algorithms using data collected in the field over a period of 24 h. The authors reported a number of false alarms ranging from 3 to 85 for different algorithms. As false alarms are relayed to an emergency response center, they result in an unnecessary use of costly resources. Besides, when an alarm is relayed to the emergency response center, the older adult has to connect with it to report his/her status. If this happens frequently, older adults are likely to display poor compliance with wearing the sensor that enables the detection of falls.

**Figure 1:**
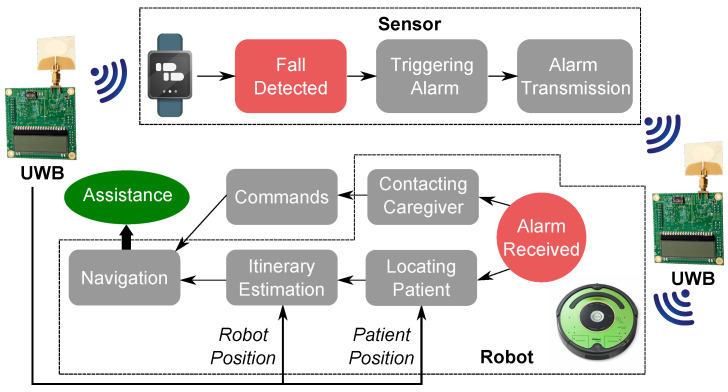
Schematic representation of the proposed fall detection system. When a fall, is detected an alarm notification is transmitted using the Decawave UWB-based system from the wearable sensor to the robot. The robot can open a communication channel with the emergency response center where personnel can manually control the robot and navigate the home environment to check the conditions of the older adult. Alternatively, the robot can autonomously navigate the environment using the UWB anchors as reference, check the conditions of the older adult, and assess if an alarm notification should be relayed to the emergency response center.

In this paper, we report the results of a follow-up study to the one that we presented in [7], where we began to explore a new approach to the design of fall detection systems that is schematically represented in Figure 1. When the system detects a fall, an alarm is relayed to a home robot, and a UWB-based tracking system is used to determine the position of the older adult and the position of the robot in the home environment. According to the configuration of the system, either a caregiver is alerted and provided with the opportunity to establish a tele-presence using the robot and navigate the home environment or the robot navigates the home environment in an autonomous manner and checks if the older adult experienced an actual fall. Assistance is then sought if necessary.

While in [7] we introduced the use of a UWB-based system for location tracking [8], we only analyzed the single node accuracy in Line Of Sight (LOS) conditions and its robustness to potential interference factors. In this manuscript, we expand upon our prior work by addressing the following issues:

- develop a machine learning algorithm to detect falls from accelerometer data collected using an IMU positioned either on the chest or on the wrist;
- assess the robustness of the alarm notification transmission system;
- evaluate the performance of the UWB-based system to locate and track a radio tag in LOS and NLOS conditions.

A first design choice for this project was that the fall detection sensor consisted of a single unit positioned on the wrist or the chest so that it could be embedded in a wrist-band or in a pendant. In addition, we aimed to avoid any alarm notification loss during the transmission between the wearable sensor and the robot. Finally, we required that the UWB-based system provided a position tracking resolution of at least 25 cm to locate the older adult and the robot in the home environment.

The material presented in the manuscript is organized as follows. In Section 2, examples of recently developed fall detection systems are presented and compared. In Section 3, we introduce the approaches and methodologies utilized in the study. Section 4 describes how the tests to characterize the proposed system were carried out during the study. Section 5 provides system performance data as well as figures of merit for its components. The results are discussed in Section 6 and conclusions are drawn accordingly. In addition to the above-mentioned material contained in the main body of the manuscript, information concerning the basic principles underlying the approaches and methodologies utilized in the study are described in the Supplementary Materials.

The main contribution of this study is the development of a new approach to address the high rate of false alarms that plagues traditional fall detection systems. The proposed solution provides a way to handle false alarms that is cost effective and does not cause any burden to the end-user. Additionally, the manuscript introduces a highly-performing machine learning algorithm to predict falls based on features that can be derived from a single tri-axial accelerometer and a machine learning algorithm to track the older adult and the robot using a UWB-based system that provides accurate results even in NLOS conditions.

## 2. Related Work

Many different approaches have been used to detect falls in older adults. Representative examples of such approaches are shown in Table 1. For a proper comparison with the proposed design, we included in this table systems that can both detect fall events and locate the subject in the home environment. These systems can be divided in three categories: vision-based, ambient sensor-based, and wearable sensor-based systems.

Vision-based systems require installing cameras in the environment and a central processing unit equipped with computer vision algorithms. These systems often raise privacy concerns. However, several techniques have been developed to protect the privacy of older adults including the development of algorithms to de-identify images by masking people’s face in the video recordings [9]. Among others, Lu et al. [10] designed algorithms to analyze video recordings obtained using surveillance cameras. The authors focused mainly on sitting down and standing up movements as older adults are at a higher risk of falling during the performance of these motor tasks. The model developed by the authors consists of an algorithm to detect when a person is in the frame and a posture recognition algorithm to detect movements associated with falls that occur during the performance of sitting down and standing up movements. De Miguel et al. [11] combined algorithms, ranging from Kalman filtering to optical flow analysis, with an algorithm designed for artificial vision. When a fall occurs, the system detects the event and sends an alarm notification message along with a picture to the caregiver. Williams et al. [12] built a multi-camera system able to localize a subject via inter-image homographies. The system combines low-power, low-resolution cameras to achieve a resolution of approximately 50 cm in determining the position of the older adult in the home environment. Dotsinsky et al. [13] developed a fall detection system combining the use of an accelerometer and monitoring cameras. To implement the proposed approach, the subject is required to wear a bracelet (an eZ430-Cronos unit by Texas Instruments, which is equipped with a tri-axial accelerometer) that is used for fall detection. When a fall event is detected, the system switches on the cameras to check the status of the older adult.

Ambient sensor-based systems exploit sensors like infrared, pressure, vibration, and radio-frequency sensors that are placed in the home environment. Data collected using these sensors is analyzed to detect fall events. These systems are unobtrusive and low-cost. However, they are typically not very accurate. For instance, pressure sensors typically provide an accuracy no better than 90% and generate a large number of false positives. Kianoush et al. [14] demonstrated that perturbations generated by the body on radio-frequency signals (i.e., signal attenuation and multipath propagation) could be used as sensing tools to identify human motion. They proposed a real-time system able to localize older adults and detect falls. Ma et al. [15] combined impulse radio ultra-wideband monostatic radar with a convolutional long short term memory neural network to detect falls. They obtained a sensitivity of about 95%, but the system could only work at room-level, becoming extremely inefficient in a house with multiple rooms. Vecchio et al. [16] proposed a fall detection method exploiting the UWB Decawave localization system (the same used in our study) and based on the analysis of the variation in the 3D position of the older adult. Unfortunately, the authors did not consider the NLOS case. Three UWB radars were exploited by Khawaja et al. [17] to build a system able to locate the subject and detect falls. The position of the older adult was estimated with an accuracy of about 30 cm without the need to use any wearable tag. Falls were detected using a Kalman filter-based approach.

The third method utilized for the development of fall detection systems is based on the use of wearable sensors. This approach is marked by high reliability and accuracy and it is the most popular approach to the development of a fall detection monitoring system. However, it requires that older adults wear a sensor. Besides, since the sensor is battery-powered, older adults have to recharge the system from time to time. Kolakowski et al. [18] developed a hybrid system that combines UWB localization and Microelectromechanical Systems (MEMS) technology. The first was used to track the position of the older adult, whereas the second gathered information concerning his/her status including the detection of falls and the characteristics of the subject’s gait. Results of experiments conducted on level ground showed a sub-meter accuracy of both static and dynamic measures. Gharghan et al. [19] built a system to detect falls in older adults equipped with an accurate procedure to identify the position of older adults in indoor environments such as health care centers or houses. Falls were identified using an accelerometer, while the tracking procedure was based on the Zigbee protocol and a neural network-based algorithm suitable for both the LOS and NLOS conditions. The sensor was placed at the waist level. Fung et al. [20] designed a fall detection and location tracking system relying on several technologies. The authors developed a wearable sensor equipped with a cellular phone, a Wi-Fi, and a Global System Mobile (GSM) module. The system sent an email and/or a SMS whenever a critical event took place. The subject’s location coordinates were derived using the Global Position System (GPS) and sent in the form of Google Maps coordinates. Potential drawbacks of this design are related to the fact that the use of the GPS module in an indoor environment could lead to sizeable errors, especially in dense urban areas.

Several commercially-available systems were developed taking inspiration from the research work summarized above. TruSense [2] offers a combination of wearable and ambient sensors. Motion sensors, placed in the rooms, and contact sensors, placed on the entrance door, allow the system to detect if the subject is at home, and if so, the sensing technology is utilized to detect if the subject experienced a fall event. Moreover, ambient sensors are utilized to monitor the room temperature and problems such as water leaks. GoLiveClip [3] is a wearable sensor connected to the subject’s smartphone via Bluetooth. The sensor is equipped with a button that the subject can press in case of need. Moreover, it is equipped with an automatic fall alarm system suitable for indoor and outdoor monitoring. The main potential drawback of this system is that it relies on the subject carrying his/her smartphone all the times, which is not necessarily common among older adults. Mynotify [21] is a wearable fall detection sensor that is available in the form of a wrist-band or a belt-clip. It is designed specifically for older adults and, like GoLiveClip, it exploits the smartphone to deliver notifications. However, in case a smartphone is not available, the system can rely on a dedicated hub.

The body of work summarized above demonstrates the tremendous interest for the development and deployment of fall detection systems. Unfortunately, all available systems generate a significant number of false alarms as discussed in the previous section. The proposed work is meant to address this important shortcoming of existing fall detection systems.

## 3. Materials and Methods

This section of the manuscript provides a description of the hardware and software components of the proposed system. The hardware components described in the following are: the home robot, the wireless sensor network, and the UWB system utilized in the study. Details are also provided about the following software components: the fall detection machine learning-based algorithm, the dataset utilized to train the fall detection algorithm, and the location tracking algorithm developed for the‘study.

### 3.1. Hardware

#### 3.1.1. Home Robot

**Figure 2:**
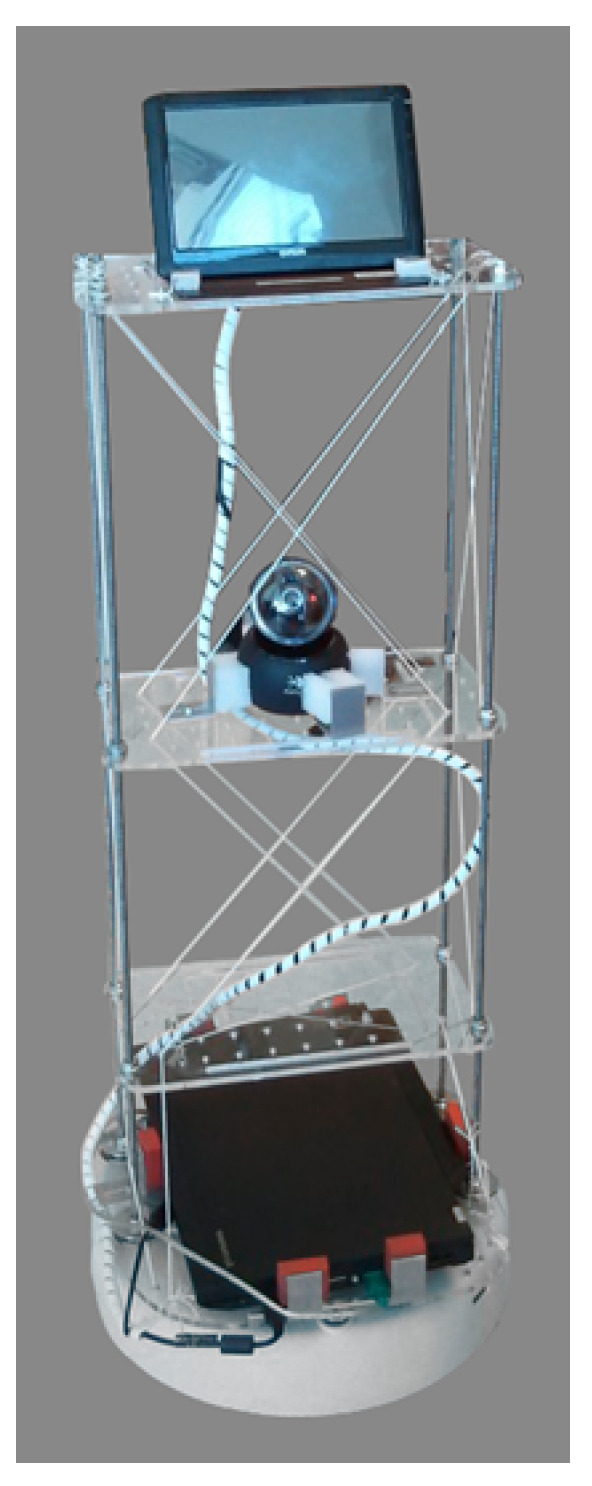
A prototype of the proposed home robot. The system consists of a IRobot Create 2, a laptop computer that was used as a control unit, a Logitech Quickcam Orbit web-camera, and a Mimo Um-720s touchscreen.

The Home Robot shown in Figure 2 consisted of four main components: an iRobot Create 2, a laptop computer that used as a control unit, a web-camera (Logitech Quickcam Orbit), and a touchscreen (Mimo Um-720s). The iRobot Create 2 (a development kit produced by iRobot) was used to enable the navigation of the environment and it provided the base of support for a shelf-like system utilized to accommodate the other components of the Home Robot.

The iRobot Create 2 was equipped with two separately driven wheels that, thanks to optical encoders, provided the traveled distance with 1 mm resolution, as well as the orientation angle with 1 degree accuracy. Each wheel could rotate independently up to 500 mm s^−1^. The touchscreen was used to enable remote interactions with the caregiver connected by video-conference. The webcam was used as the “eyes” of the caregiver. All these components required a processing unit, which we implemented using a laptop computer. The robot was connected to the laptop computer via a serial port that enabled communication with the following characteristics: baud rate = 57,600, data bits = 8, and stop bit = 1. Software was developed and installed on the laptop computer to control the movements of the robot, transmit commands through the serial port and receive data from the sensor. The software also managed a video conferencing module and enabled remote control of the robot. Thanks to a simple interface, the caregiver could control the robot and the orientation of the web-camera.

#### 3.1.2. Wireless Sensor Network

A Wireless Sensor Network (WSN) was built using the Decawave Real-Time Localization System (RTLS) [22,23]. The WSN was used to track the position of the subject and of the robot in the home environment, facilitate the navigation of the home environment by the robot, and relay alarm notification messages to the caregiver. Specifically, we used the TREK1000 [23] system, which is equipped with four EVB1000 evaluation boards based on the DW1000 IC transceiver. TREK stands for Two Way Ranging (TWR) RTLS IC Evaluation Kit. Each board was programmable and could work both as an anchor as well as as a radio tag. Three different modalities of use could be selected: tracking, geo-fencing, and navigation mode. In the tracking mode, the position of radio tags was estimated using fixed anchor units as reference. In the geo-fencing mode, the system was set to determine if radio tags left or entered a specific area, typically defined as the distance from a set anchor unit. In the navigation mode, the system functioned in a way similar to the tracking mode. However, in the navigation mode, the position of the processing unit (laptop computer) was determined using the anchor units as reference. The system was tested using two radio tags: one connected to the laptop computer and one placed in the laboratory as if it was worn by the subject.

**Figure 3:**
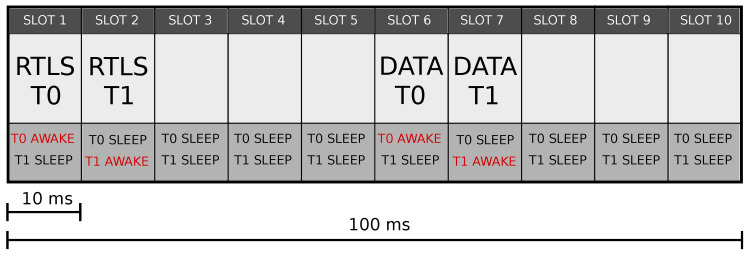
Superframe structure with the division in 10 time slots.

The WSN supported the exchange of messages between units while enabling the real-time localization of the radio tags. The WSN communication protocol between units (e.g., T0 and T1) relied on the Time-Division Multiplexing (TDM) protocol, in which the exchange of messages occurs in a time frame called superframe, Figure 3. The superframe corresponds to a TDM cycle composed of 8 time-slots for the TWR protocol and 2 time-slots dedicated to the anchor communication for a total of 10 time-slots of equal duration. Only one radio tag per slot is allowed to transmit over the UWB channel, while the other radio tags are in idle mode. The Anchor0 is the TDM master and is responsible for assigning and maintaining radio tags in their time-slots.

Two types of time-slots are available to keep the communication protocol modular and scalable: the RTLS time-slots and the DATA time-slots. In the RTLS time-slots, the owner tag set the communication using the TWR protocol. The chosen data rate provided the minimum duration of the time-slot: for instance, for 110 kbps, it was 26 ms, whereas for 6.81 Mbps, it was 2.25 ms. The data rate for the application herein presented was set to 6.81 Mbps which allowed us to have as many samples as possible. Three types of messages could be sent using a RTLS slot: poll, answer, and final message. The poll was sent by the radio tag to start a range (i.e., distance) measurement. The answer message was sent by the anchor in response to a poll, while the radio tag sent the final message after receiving the anchor’s response message. These three messages carried time information required to estimate the time of flight (ToF). In the DATA time-slots, the owner tag could send messages to Anchor0 and query for incoming messages from other radio tags. The message was composed of two parts: the Tag Query and the Anchor Response. The Data Message Tag Query was sent by the radio tag to communicate to other radio tags and retrieve messages. The anchor sent the Data Message Anchor Response in response to a query. The general message format for a data frame was the IEEE 802.15.4 standard encoding.

The WSN infrastructure supported the transmission of alarm notification messages. In a hypothesical scenario in which a subject experiences a fall, T0 would receive the alarm signal triggered by the sensor and transmit it to the robot. In return, T1 would acknowledge receipt of the alarm notification message and wake up the robot. Thus, two TWR processes and two data messages per superframe would be required. Consequently, 4 time-slots, 2 RTLS, and 2 DATA slots would represent the minimum size of a superframe. Since the total TDM cycle was composed of 10 time-slots, by using only 4 of them, almost 60% of the required energy was saved, increasing the battery lifetime. it is worth noting that a superframe turnover frequency of 10 Hz would lead to a RTLS application suitable for a robot that moves at a maximum speed of 50 cm s^−1^; this means a sample every 5 cm.

#### 3.1.3. Location Tracking Using UWB Radio Systems

Multipath is a propagation phenomenon that leads to a radio signal reaching the receiving antenna via multiple paths. Typical causes of multipath are reflection and refraction caused by walls and objects in the home environment. Radio signals affected by multipath result in constructive and/or destructive interference. The latter causes fading [24], namely an attenuation of the radio signal. The interference phenomenon makes it challenging to identify the radio signal that travels in LOS conditions as it reaches the receiver [25]. UWB is a robust technology [26,27] from this point of view because the large bandwidth [28,29] used by UWB radio systems results in high time resolution [30] (i.e., short pulses) thus enabling the receiver to identify components of the radio signals that are the result of LOS propagation (see Supplementary Materials Section 1.1 and 1.2 for additional information regarding UWB and the localization problem). This property makes UWB radio systems particularly suitable not only for location tracking applications in indoor environments but also for short distance estimation [31], and, more generally, for all architectures relying on a time-coded or quasi-digital approach [32,33,34], which in turn allows for the design of very small and simple [35,36] low-power read-out-circuits [37].

**Figure 4:**
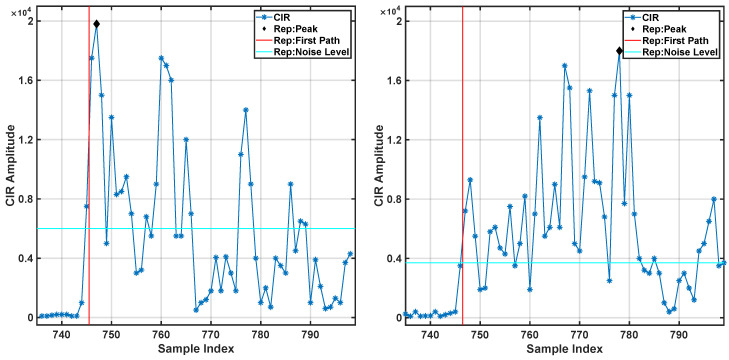
CIR example of Line of Sight (**left**) and No Line of Sight (**right**) condition.

When propagation cannot occur via direct LOS, the only pulses that reach the receiving antenna do so via multipath propagation. This translates in a longer ToF and, consequently, in an overestimation of the transceiver’s distance from the receiver. Hence, it is apparent that one should mitigate the effects of the NLOS propagation. Channel Impulse Response (CIR) [38] is a diagnostic tool available as part of the Decawave system to address this problem. It is defined as the magnitude of the received energy by the transceiver unit in time. By analyzing the CIR time-series, one can identify radio signals that propagate in LOS conditions and radio signals that propagate in NLOS conditions. Three specific features of the CIR could be useful in identifying if a radio signal reached the anchor in LOS or NLOS conditions. These features are: the Received Signal power Level (RSL), the Rise Time (RT), and the First Path Gain (FPG). It is worth noting that the RSL is an estimate of the CIR total power and can be computed with the Equation:

(1) $$RSL\left(\mathrm{dB}\right)=10\times log_{10}\frac{C\times 2^{17}}{N^{2}}-A$$

where *C* is the channel input response power, *N* is the preamble accumulation count, and *A* is a predefined constant (115.72 for a pulse repetition frequency of 16 MHz and 121,72 for 64 MHz). Intuitively, anchors affected by NLOS would display a lower RSL value due to the signal attenuation associated with the presence of the obstacle that obstructs the LOS. The RT parameter is defined as the difference between the time of arrival of the maximum peak of the CIR time-series and the first peak above the threshold noise level. In LOS conditions, the two are expected to coincide because the impulse traveling through the most direct path is the least attenuated and the first to be detected. Finally, the FPG parameter is the ratio between the first peak amplitude and the CIR maximum. This feature varies in the range between 0 and 1. Lower values are associated with NLOS conditions, where the maximum of the CIR is due to multipath transmission, and the first peak is associated with the packet that is attenuated by the presence of an obstacle. Example of data collected in LOS and NLOS conditions are shown in Figure 4.

### 3.2. Software

#### 3.2.1. Fall Detection Machine Learning-Based Algorithm

Our work expanded on the study carried out by Ozdemir [39] by first attempting to improve the accuracy of the detection of fall events by relying on data collected using nine-axis units and then assessing if it is possible to obtain comparable results by using data features from a single tri-axial accelerometer. The latter would relax the sensor hardware constraints, reduce cost and increase battery life. In our analysis, we focused on using the units positioned on the wrist and the chest as they could be embedded in a wrist-band or a pendant, respectively, two form factors that are very popular and typically accepted by most end-users.

The time series from the sensor units were high-pass filtered using a 6th order type 2 Chebyshev filter with cut-off frequency of 0.3 Hz. Next, 15 different data features were extracted for each axis of the accelerometer, gyroscope, and magnetometer sensors. We derived data features in the time and frequency domains. In the time domain, we computed the mean, maximum, range, standard deviation (SD), root mean square (RMS) value, skewness, entropy and kurtosis o fht etime series. In the frequency domain, we computed the dominant and median frequency as well as the relative energy of the dominant frequency to the total energy of the time-series. In addition, to detect abrupt changes in the time series, we estimated the maximum, SD, RMS, and range of the derivative of the time-series. This procedure led to a total of 135 data features per unit that were z-score normalized and ranked using the ReliefF algorithm. The ReliefF feature selection algorithm was developed by Kononenko et al. [40]. It is based on computing the importance of each data features, by randomly choosing instances in the dataset and evaluating if the considered data feature is marked by similar values for data points of the same class and different values for data points of different classes. To select the optimal number of data features to feed the classifier, different approaches can be used. Because the dimensionality of the data feature space for the problem at hand was relatively small, we opted for training multiple classifiers starting with 5 data features and adding one data feature at the time according to their ranking until a maximum of 100 data features was reached. We anticipated that the model’s performance would have reached an asymptotic value when the data features incrementally added during the above-described procedures did not contribute any additional information. Whereas previous studies suggested that satisfactory results could be obtained using relatively simple classification algorithms [41], the results obtained by applying simple classification algorithms to the above-listed set of data features were not satisfactory. Hence, we opted for using a regression implementation of a random forest model (number of trees = 100, min leaf size = 2). This algorithm can be easily implemented on a wearable sensor, it is robust to overfitting, and does not require normalization of the data features. The validation of this model was achieved using the Leave One Subject Out (LOSO) cross-validation technique first training the algorithm first with the data features extracted from all the sensor time series (i.e., recorded using the accelerometer, gyroscope and magnetometer units) and then using only the 45 features derived from the accelerometer time series.

#### 3.2.2. Dataset of Fall Events

To develop the machine learning-based algorithm for fall detection described above, we used a dataset previously collected and made publicly available by Ozdemir et Barshan [41]. The dataset consists of 3,060 instances of 36 different movements repeated 6 times by 17 volunteers. The movements included 20 simulated falls and 16 activities of daily living. During the experiments, the subjects wore six MTw units with embedded tri-axial accelerometers, gyroscopes, and magnetometers. The output range of these sensors was set to ± 120 ms^−2^, ± 1200°/s and ±1.5 Ga, respectively. The sensors were positioned on the following body segments: head, chest, waist, right wrist, right thigh, and right ankle. From the data gathered using each accelerometer unit, we derived the above-described data features over time windows of 4 s.

The dataset was used to test different machine learning-based algorithms for the detection of fall events [41]. The authors were able to obtain a good fall detection rate by using a k-NN classifier. This algorithm provided accuracy, sensitivity, and specificity all above 99%. In an additional study [39], the data was analyzed to compare the accuracy of fall detection when using sensors positioned on different body segments, focusing in particular on the algorithm performance when a single unit was utilized. An accuracy of 94.9% was obtained when using a single sensor positioned on the right wrist. The results were obtained using a 10-fold cross-validation. This approach often leads to overestimating the model’s performance since repetitions belonging to the same subject are used for training and testing simultaneously. To avoid this problem, we opted instead for using the LOSO cross-validation technique, an approach in which the data of one subject is iteratively held out, the model is trained, and testing is carried out on the held-out data.

#### 3.2.3. Location Tracking Algorithm

**Figure 5:**
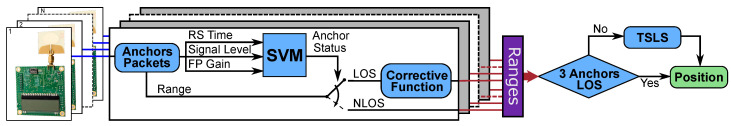
Schematic representation of the location tracking algorithm. The estimated range (i.e., distance) values of the anchors in NLOS conditions are used to estimate the position if the radio tag only if less than three anchor units have direct LOS to the radio tag.

Figure 5 provides a schematic representation of the algorithm utilized to derive estimates of the position of the subject and the position of the robot in the home environment based on data collected using the Decawave units. The *RS Time*, *Signal Level*, and *FP Gain* parameters derived from different anchor units were fed to a Support Vector Machine (SVM) based classification algorithm to determine if the data was gathered in LOS or NLOS conditions. Specifically, we used a SVM with a Radial Basis Function kernel and a C-parameter value equal to 1. To generate the training set and the test set to build and characterize the machine learning algorithm, we collected a dataset of 6600 samples, with 4950 data points collected in LOS conditions and 1650 data points collected in NLOS conditions. The data was collected using different relative positions of the anchor unit and the radio tag as well as of the object obstructing the LOS.

If the SVM-based classifier determined that the data was collected by a specific anchor unit in LOS conditions, then the estimated range value (i.e., distance from the anchor) was multiplied by a corrective function that accounted for the baseline distortion as measured during bench tests. If three or more anchor units gathered data in LOS conditions, the standard trilateration approach was applied. If less than three anchor units gathered data in LOS conditions, we implemented the Taylor Series-based Least Square Algorithm (TSLS) proposed by Yu et al. [42] (additional details in Section 1.3 of the Supplementary Materials) to mitigate the effect of the estimation error associated with the NLOS condition on the trilateration algorithm. The position estimates were then smoothed using a moving average window of 10 samples. The model was implemented using Matlab 2019.

## 4. Experimental Procedures

### 4.1. WSN Alarm Notification Transmission

Every time the machine learning algorithm detects a fall, the wireless sensor network should relay an alarm notification message to the home robot. To achieve this goal reliably and effectively the system has to be marked by high robustness (i.e., no alarm notification message should be lost) and rapid handling of notification messages (i.e., a short time should elapse between the time when the alarm notification message is generated and the time when it is delivered).

The superframe composition described in Section 3.1 is designed to send data efficiently from T0 (i.e., the tag worn by the older adult) to T1 (i.e., the tag positioned on the robot). The oversized time-slot duration grants to the radio tag the possibility to re-query the anchor unit in case an error occurs the first time the alarm notification message is sent. The estimated time between a query and its response is less than 3 ms; therefore, the tag has 7 ms to recover from the occurred error.

To test the reliability of the protocol, T0 and T1 were connected to a computer that simulated the random generation of alarm notification messages. Each message was randomly generated within a separate interval of 100 ms. The computer checked the communication and determined the elapsed time from when the alarm notification message was generated to when it was received, with sub-millisecond precision. To test the WSN alarm transmission, we generated approximately 400,000 alarm messages over a simulated time span of 15 h.

### 4.2. Position Tracking Experiments

We performed a series of experiments to analyze the UWB tracking system’s behavior under different conditions, starting with static measurements, with LOS between the anchor units and the radio tag, and ending with dynamic tracking, with an object obstructing the transceivers’ LOS.

Four different sets of experiments were carried out:

- 1D static measurements in LOS conditions, to analyze the ability of the system to estimate the distance between individual anchor units and the radio tag;
- 2D static measurements in LOS conditions, to analyze the performance of the system in estimating the radio tag’s coordinates;
- 2D static measurements in NLOS conditions, to analyze the ability of our algorithm to compensate for the error introduced by obstacles obstructing the LOS;
- 2D dynamic measurements in NLOS conditions, to simulate the conditions in which we would anticipate that the home robot would operate in a real-life deployment.

#### 4.2.1. 1D Static Measurements in LOS Conditions

A one-dimensional study was performed using one anchor unit and one radio tag. Maintaining the anchor unit in a fixed position, estimated range (i.e., distance) values were recorded by moving the radio tag along a straight line by increments of 25 cm from a position close to the anchor unit to a position 10 m away from the anchor unit.

Four different antenna orientations were tested. In the first set of tests, the antennas were directed toward each other (orientation 1). In the second set of tests, the antenna of the radio tag was rotated 90° clockwise in order to be perpendicular to the antenna of the anchor unit (orientation 2). The third configuration that we tested had the antenna of the radio tag directed toward the anchor unit (orientation 3), while the antenna of the anchor unit was rotated 90°. Finally, in the last series of tests, both antennas were rotated 90° (orientation 4). This allowed us to assess how the range estimates were affected by the orientation of the antennas and by the relative distance between the anchor unit and the radio tag.

#### 4.2.2. 2D Static Measurements in LOS Conditions

2D static measurements in LOS conditions provided a reference value for tests later performed in NLOS conditions and tests performed in dynamic conditions (i.e., while moving the radio tag). In fact, static measures in LOS conditions are gathered in what are theoretically the best conditions of operation of the system. During these tests, we assessed not only the accuracy of the system in different conditions but also we evaluated if increasing the number of anchor units could improve the accuracy of the radio tag tracking. For this reason, we tested two different configurations, using eight and four anchor units, respectively. Since the system was designed to work with a maximum of four anchor units, we used the two available pulse transmission frequencies (i.e., 3.6 GHz and 6.5 GHz) simultaneously to operate two sets of four anchor units. Four of the anchors sent the range estimates using the first channel, while the remaining four anchors used the second channel. The radio tag was programmed to switch between the two channels, so that data could be collected using both sets of four anchor units. A measurement grid was used to position consistently the anchor units and the radio tag.

#### 4.2.3. 2D Static Measurements in NLOS Conditions

When the robot navigates the home environment, furniture and other objects would at times obstruct the LOS and hence data would be gathered by the anchor units in NLOS conditions. Increasing the number of anchors together with choosing their position appropriately could reduce the number of data points gathered in NLOS conditions and hence one could solely rely on data points collected in LOS conditions. However, for an effective deployment of the technology, it is desirable that strategies suitable to track the older adult as well as the robot in NLOS conditions be available. In fact, NLOS conditions are most likely to occur in a real-life deployment of the technology. Hence, a batch of static tests in NLOS conditions were carried out to evaluate the estimation error associated with the presence of objects obstructing the LOS.

For these measurements, three anchor units were kept in a fixed position while the radio tag was moved according to a measurement grid. As the obstacle to obstruct the LOS, we used a metallic case (dimension 0.5 m × 0.3 m × 0.7 m) positioned 50 cm away from one of the three anchors. This is a challenging case for the tracking system because, due to the object’s material properties, the radio wave is entirely reflected. In addition, the proximity to the anchor unit dramatically obstructs the LOS. The measurements were repeated multiple times with different anchor units in NLOS conditions and in three different locations. To collect CIR data, we modified the Decawave firmware so as to add at the end of the packet the information of interest. For each anchor unit, the following three parameters were stored: signal level, rising time, and gain. These triplets composed the dataset used to train a SVM-based algorithm that we designed to predict if the data was collected in LOS or NLOS conditions.

#### 4.2.4. 2D Dynamic Measurements in NLOS Conditions

2D dynamic measurements were gathered to simulate the conditions in which the anchor units would track the radio tag positioned on the robot (as the robot navigates the home environment) in a real-life deployment of the system.

Data was collected using the anchor configuration shown in Section 5.3.5 (Anchor 0 in [1.3, 3.4], Anchor 1 in [1.3, −1.4], and Anchor 2 in [−1.4, –1.4]). As the robot moved, its position was tracked using a camera-based motion tracking system relying on infrared cameras and reflective markers that we positioned on the robot. The VICON [43] system (Oxford Metrics Group, Cambridge UK) was used to collect this data. Simultaneously, data was collected with the Decawave kit and used to estimate the position of the radio tag by relying on the algorithms described above. Six different trajectories were tested including linear and curvilinear paths while objects obstructing the LOS were set in different positions.

## 5. Results

This section summarizes the results obtained with the proposed fall detection algorithm, the outcomes of the WSN performance tests, and the characterization of the UWB-based position tracking system developed in the study.

### 5.1. Fall Detection Algorithm

**Figure 6:**
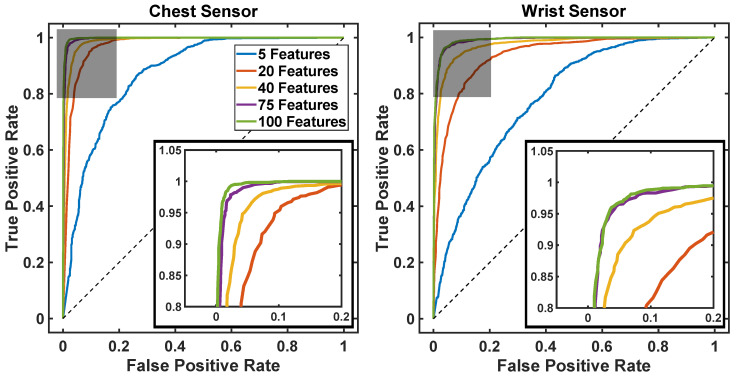
ROC curves for different numbers of data features used as input to the proposed machine learning-based algorithms.

Figure 6 shows the ROC curves derived from the data collected using a sensor on the chest (left panel) and on the wrist (right panel). These results were obtained by using data features derived from the none-axis units, i.e. the accelerometer, gyroscope and magnetometer time series. Different ROC curves are shown to highlight the effect of choosing different numbers of data features.

**Figure 7:**
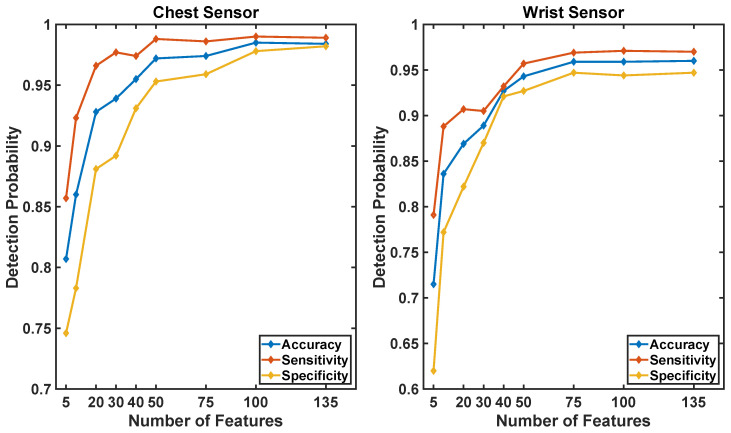
Algorithm performance as a function of the number of data features used as input to the proposed machine learning-based algorithms.

Figure 7 shows the accuracy, sensitivity, and specificity of the machine learning algorithm as a function of the number of features used by the model. The sensitivity and specificity values plotted in this figure were derived by choosing the operating point of the ROC curves shown in Figure 6 associated with maximum accuracy.

**Figure 8:**
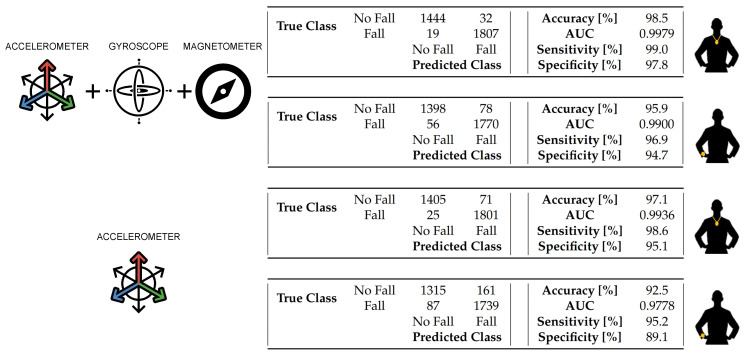
Performance of the model as a function of the position of the sensor (wrist vs chest position) used to derived data features utilized as input the proposed machine learning-based algorithms. Results are shown for analyses based on data collected using nine-axis units (i.e., accelerometer, gyroscope, and magnetometer time series) and for analyses based on data collected using only the accelerometer time series.

The results show that data collected using a sensor positioned on the chest provides better fall detection estimates than data collected using a wrist unit (Figure 8). This result is consistent with previous literature suggesting that a pendant is more suitable than a wrist unit to monitor falls in older adults [39]. Although we used the LOSO cross-validation technique, which is known to be a more conservative approach than the k-fold cross-validation technique (and hence would typically provide results displaying lower values of sensitivity and specificity), the accuracy of the algorithm proposed in this study turned out to be slightly higher than the accuracy displayed by previously proposed algorithms that were evaluated on the same dataset but using a k-fold cross-validation technique [39].

### 5.2. WSN Performance

Table 2 shows the results of the tests carried out to characterize the WSN performance. We generated approximately 400,000 alarm notification messages and transmitted them over a simulated time intervals of 15 h. All the alarm notification messages transmitted by the T0 radio tag unit were received by the T1 unit and all within 106 ms. T0 had to perform a re-query 38,399 times (10.01% of the times). Only 12 times this was because of a timeout affecting the receiving unit response (i.e., the unit did not reply). The remaining 38,387 re-query instances were caused by a message using Slot 6. T1 performed a re-query 34 times due to a unit reply timeout. Neither T0 nor T1 had to re-query more than once.

The first row in Table 2 contains the number of alerts per time-slot sent by T0, while the second and third rows show the minimum and the maximum time elapsed from when the alarm notification message was triggered to when an action was taken. The fastest message took only 7 ms to reach the receiving unit, while the slowest message was delivered in 106 ms.

### 5.3. Position Tracking

In the following, we summarize the results obtained during the first sets of experiments that we carried out to characterize the UWB-based position tracking system utilized in the study.

#### 5.3.1. 1D Static Measurements in LOS Conditions

**Figure 9:**
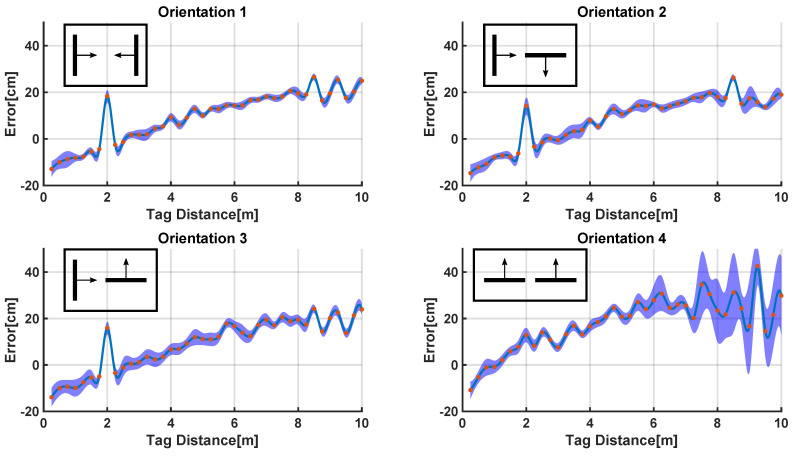
Error affecting the estimates of the distance between the anchor unit and the radio tag for different orientations of the antennas. The error is higher for orientation 4 where both the antenna of the anchor unit and the antenna of the radio tag are aligned. The red dots represent the tested data points. The blue areas represent the variance of the estimates.

Figure 9 shows the estimation error associated with all four antenna orientations as a function of the distance between the radio tag and the anchor unit. The system underestimated the distance between the radio tag and the anchor unit for values smaller than 2 m, while it overestimated such distance for values greater than 2 m.

To compensate for the trend shown in Figure 9, a corrective function was derived and applied to the data as shown in Equation (2), where *range* and *f*() are the range (i.e., distance) estimate and the corrective function, respectively, while *range’* is the corrected value.

(2) $$range^{\prime }=range\;\cdot \;f\left(range\right)$$

Such corrective function compensated, at least in part, for the estimation error affecting the computed range (i.e., distance) values and was applied to the data collected during all the 2D experiments.

#### 5.3.2. 2D Static Measurements in LOS Conditions

**Figure 10:**
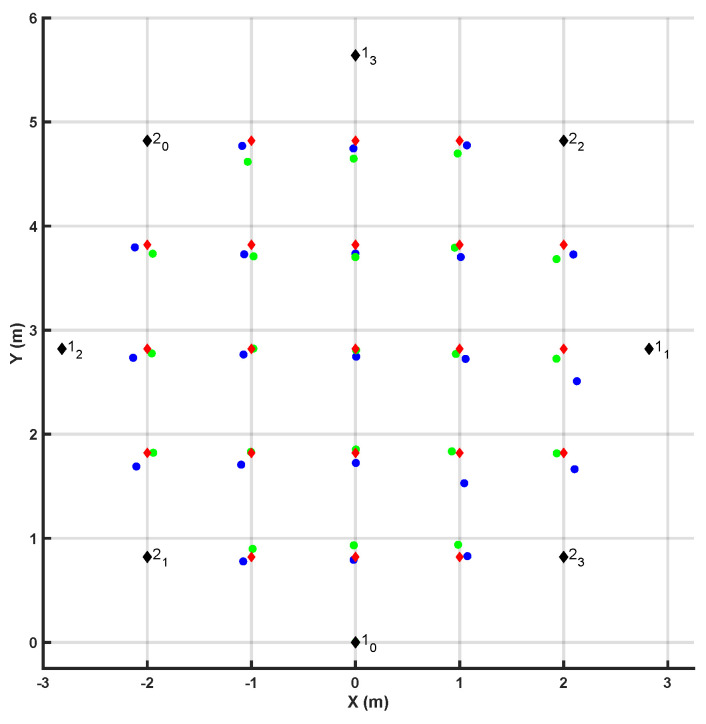
Comparison of the results obtained with vs. without correction algorithm when eight anchor units were used to derive the position of the radio tag. The black markers represent the coordinates of the anchors. The red markers represent the actual position of the radio tag for all tested positions, the blue markers represent the estimated positions before the correction algorithm was applied, and and the green markers represent the coordinates after the correction algorithm was applied.

In order to investigate how the number of anchor units affects the accuracy of the range estimates, eight anchor units were positioned as shown in Figure 10. Two sets of four anchor units were utilized as described above. Anchors labeled *1${}_{x}$* were part of the first set of units, whereas anchors labeled *2${}_{x}$* were part of the second set of units.

Using the above-stated configuration, we derived the static error characteristics in LOS conditions. The trilateration method was used to estimate the position of the radio tag first by using all available estimates and then by using only estimates derived using the first set of four anchor units.

Furthermore, we evaluated the effects of the above-mentioned corrective function on the estimates derived from 2D static measurements in LOS conditions. Table 3 shows the average error (calculated as the Euclidean distance between the actual and the estimated position of the radio tag) at the edges vs. the middle of the area covered by the anchor units when using four vs. eight anchor units and with vs. without using the above-mentioned corrective function. Anchors labeled *1${}_{x}$* were part of the first set of anchor units, whereas those labeled *2${}_{x}$* were part of the second set of units.

Figure 10 highlights the benefits of using the corrective function as the estimates after the corrective function was applied are generally closer to the actual position of the radio tag than the estimates prior to applying the corrective function. However, Table 3 shows that the benefits of the corrective function do not equally apply to the instances in which the radio tag unit is in the middle of the area covered by the anchor units and the instances in which the radio tag unit is at the edges of such area.

#### 5.3.3. 2D Static Measurements in NLOS Conditions—NLOS Detection

The SVM-based detection algorithm of LOS vs. NLOS is an important element of the proposed system. Estimates derived from data collected in NLOS conditions are unreliable and an error mitigation strategy is necessary in order to obtain acceptable estimates of the radio tag position. The first step toward implementing an error mitigation strategy is clearly the identification of data points gathered in NLOS conditions. The performance of the SVM-based detection algorithm is shown in Table 4. The confusion matrix was derived using a 10-fold cross-validation and shows the high accuracy of the classification algorithm.

#### 5.3.4. 2D Static Measurements in NLOS Conditions and NLOS Mitigation

**Figure 11:**
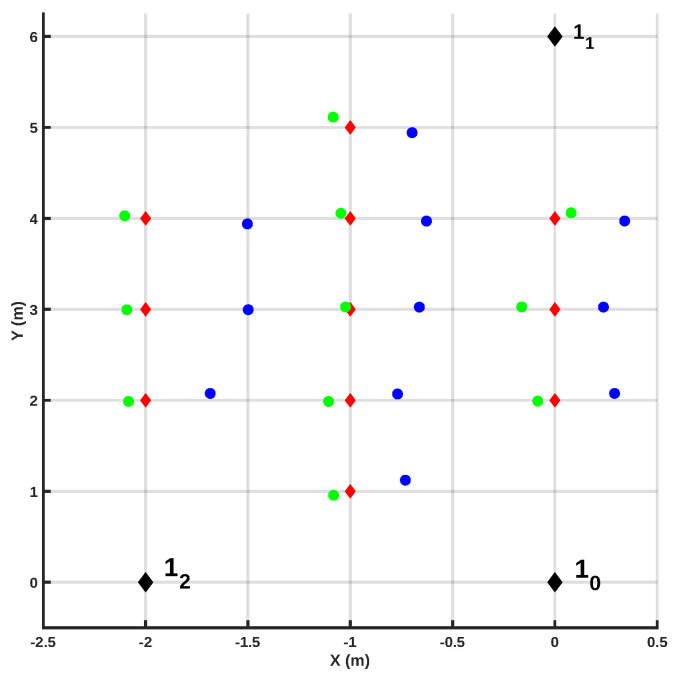
TSLS correction for static measurements, Anchor 2 in NLOS.

The LOS/NLOS classifier allows one to identify the data points collected in NLOS conditions. Still, if at least three anchor units gather data from the radio tag in LOS conditions, it is possible to apply the trilateration algorithm to the range estimates derived from the data collected using these anchor units. However, when less than three anchor units gather data in LOS conditions, it is necessary to implement an error mitigation algorithm to the estimates derived from data collected in NLOS conditions. Specifically, the proposed system applied the TSLS algorithm mentioned above. An example of the benefits of this approach is shown in Figure 11. These results were obtained using three anchor units. When the proposed mitigation approach was applied, the average estimation error was more than halved, decreasing from 0.33 ± 0.06 m to 0.10 ± 0.03 m. Albeit the TSLS algorithm does not compensate completely for the estimation error associated with the NLOS condition, the residual error (after the application of the proposed mitigation approach) satisfies the requirements of the problem at hand.

#### 5.3.5. 2D Dynamic Measurements in NLOS Conditions

**Figure 12:**
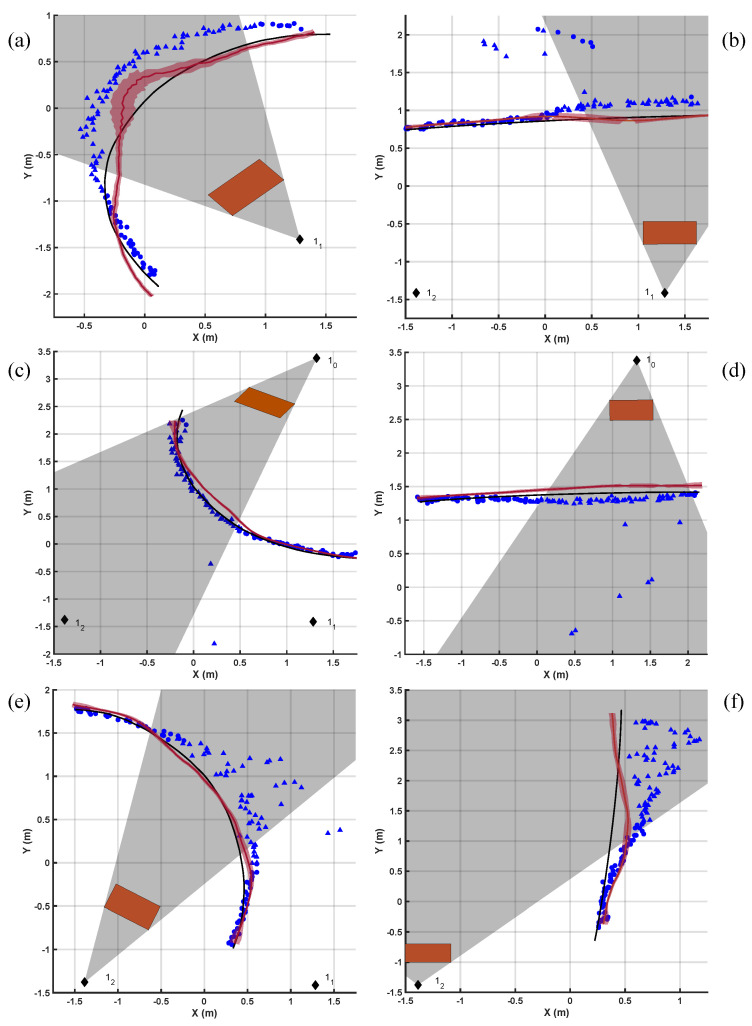
The six subfigures (**a**–**f**) represent dynamic tests, with the robot moving along six different trajectories. (**a**,**c**,**e**) represent circular trajectories, while (**b**,**d**,**f**) are linear ones. The black line represents the position measured by the Vicon system, while the blue markers are the coordinates estimated originally by the Decawave kit. The marker shapes are triangular if the SVM classified the point as collected in NLOS conditions or circular when LOS was predicted. The red line in the graph is the corrected trajectory with the shaded area showing the variability computed as standard deviation within the moving average window. The black diamonds show the positions of the anchors while the red rectangles represent the position of the obstacles. Finally, the grey area is a rough estimate of the field of view in which the anchor operates in NLOS conditions.

**Figure 13:**
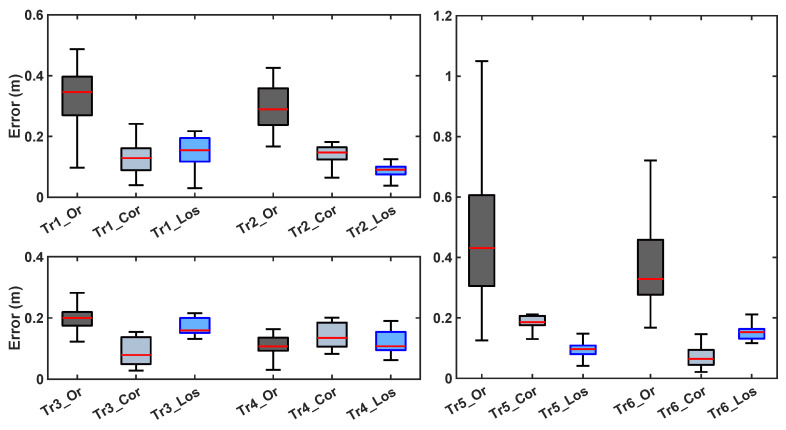
NLOS error distribution before (dark grey boxes) and after the correction (light grey boxes) for the six different trajectories. The light blue boxes represent the error for the LOS points. For the graph clarity, the 6 trajectories have been divided into three subplots with different ranges. Starting from the top left, counterclockwise, are shown the error distributions of the trajectories from one to six, which correspond to the ones in Figure 12 from (a–f).

Figure 12 show the results of the dynamic tests carried out during the study. These tests aimed to assess the accuracy of the estimates of the robot position in conditions that closely mimicked those that one would anticipate in a real-life deployment of the system. Estimates of the robot position derived using a camera-based motion tracking system (VICON, Oxfor Metrics Group, Cambridge UK) were used as reference (i.e., gold standard). Figure 13 shows a summary of the estimation error for different trajectories with vs. without the proposed estimation error mitigation strategy, hence demonstrating its benefits when applied to data collected in dynamic conditions.

## 6. Discussion and Conclusions

The machine learning-based algorithm developed in the study provided us with results more accurate than simpler algorithms previously applied by others to the same dataset. It should be emphasized that, despite the fact that we obtained high sensitivity and specificity in the detection of fall events, the likelihood of false alarms associated with the proposed algorithm is incompatible with the field deployment of a system solely relying on a fall detection sensor combined with the proposed algorithm. This observation further justifies the development of the proposed system leveraging the use of a home robot to improve the management of false alarms. Interestingly, when using the home robot to address the problem of false alarms, one could envision utilizing a wearable sensor for fall detection equipped solely with a tri-axial accelerometer rather than a nine-axis unit. In fact, the machine learning-based algorithms relying solely on data features derived from the accelerometer time series presented similar values of sensitivity to those relying on data collected using nine-axis units. Although the latter displayed better performance in terms of accuracy and specificity, the effect of the increased rate of false alarms associated with using only the accelerometer time series would only result in requiring that the home robot checks more frequently the status of the older adult.

The WSN system utilized in the study was shown to be suitable to relay alarm notification messages to the home robot. Our tests confirmed the robustness of the communication protocol as no message was lost out of the approximately 400,000 alarm notification messages that we generated over a simulated time interval of about 15 h. In addition, thanks to the composition of the data packets and the organization of the time-slots utilized by the communication protocol, alarm notification messages were delivered with a maximum latency of 106 ms, thus ensuring a fast response to fall events.

We identified as an important design requirement that the error affecting the radio tag position estimates generated by the system be smaller than 20 cm. Since the system is meant to operate in the home environment, this design requirement should be met when data is gathered not only in LOS conditions but also in NLOS conditions. This is because, in the home environment, one would expect that the furniture and other objects would obstruct the LOS. To achieve this objective, we proposed an error mitigation approach based on two main steps: (1) identify if the data was collected in LOS vs. NLOS conditions, and (2) apply the appropriate estimation procedures according to whether the available data points were derived from measures gather in LOS or NLOS conditions.

The classification algorithm based on a SVM model that we proposed to detect the LOS/NLOS conditions performed satisfactorily. However, when observing the results in Figure 12, it is apparent that the proposed algorithm might fail when it is applied to data points collected in very challenging conditions (i.e., combinations of trajectory characteristics and relative position of the anchors that make the disambiguation task rather challenging). Nonetheless, the average estimation error obtained once all the proposed error mitigation strategies were applied and the robot trajectories were estimated was generally satisfactory. It should be noted that, as observed in previous studies [44,45], the residual estimation error (i.e., after implementing the error mitigation strategy) is not the same across data points within the area covered by the anchor units. Future work should address error mitigation strategies accounting for this observation.

Overall, the position tracking algorithm provided accurate reconstructions of the movement trajectories tested in the study. Sang et al. [46] also studied this problem using the same UWB kit utilized by our research team. The authors were able to obtain accurate location estimates when at least three anchor units collected data in LOS conditions. In our study, we implemented a more robust approach that relied on a TSLS-based algorithm to generate accurate estimates of the position and of the trajectory of displacement of the radio tag.

In conclusion, this study provides evidence of the feasibility of augmenting traditional fall detection systems by using UWB-based position tracking and a home robot to mitigate the effects of false positives that affect conventional systems. Although this paper is focused on the detection of falls, this is just one of the many possible applications of the proposed approach, and the concept of combining technologies to address the effects of false positives can be easily extended, with the proper adjustments, to other domains.

## Figures and Tables

**Table 1:** Examples of recently developed fall detection systems.

	Name	Class	Sensor Type	Location
Research Systems	Lu et al. [10]	vision-based	cameras	indoor
	De Miguel et al. [11]	vision-based	cameras	indoor
	Williams et al. [12]	vision-based	cameras	indoor
	Dotsinsky et al. [13]	vision-based	cameras	indoor
	Kianoush et al. [14]	ambient-based	radio-frequency antennas	indoor
	Ma et al. [15]	ambient-based	UWB	indoor
	Vecchio et al. [16]	ambient-based	UWB	indoor
	Khawaja et al. [17]	ambient-based	UWB	indoor
	Kolakowski et al. [18]	wearable-based	UWB + MEMS	indoor
	Gharghan et al. [19]	wearable-based	accelerometer + Zigbee WSN	indoor
	Fung et al. [20]	wearable-based	GPS + GSM + smartphone	indoor/outdoor
Commercial Systems	TruSense [2]	wearable-based	wearable + motion +contact sensors	indoor/outdoor
	GoLiveClip [3]	wearable-based	wearable sensor + smartphone	indoor/outdoor
	MyNotify [21]	wearable-based	wearable sensor + smartphone/hub	indoor

**Table 2:** WSN alarm notification message benchmark data.

	SLOT 1	SLOT 2	SLOT 3	SLOT 4	SLOT 5	SLOT 6	SLOT 7	SLOT 8	SLOT 9	SLOT 10
# of messages	38,387	38,359	38,375	38,178	38,945	37,955	38,366	38,311	38,663	38,002
min time [ms]	57	67	77	87	97	7	17	27	37	47
max time [ms]	66	76	86	96	106	16	26	36	46	56

**Table 3:** Estimation error for radio tags positioned in the middle of the area covered by the anchor units vs. the edges of such area when using eight vs. four anchor units.

	8 Anchors		4 Anchors	
	Correction	No Correction	Correction	No Correction
**Error in the middle [m]**	0.0552	0.1015	0.0517	0.1305
**Error at the edges [m]**	0.111	0.1180	0.093	0.196

**Table 4:** Confusion matrix of the LOS/NLOS classifier.

True Class	LOS	4921	1
	NLOS	8	1670
		LOS	NLOS
		**Predicted class**	

## Supplementary Materials

The following are available at https://www.mdpi.com/1424-8220/20/18/5361/s1.

## References

[1] Nari M., Suprapto S., Kusumah I., Adiprawita W. A simple design of wearable device for fall detection with accelerometer and gyroscope. Proceedings of the 2016 International Symposium on Electronics and Smart Devices (ISESD).

[2] TruSense. https://www.mytrusense.com/.

[3] GoLiveClip. https://www.goliveclip.eu/.

[4] Telepresence Robot. https://telepresencerobots.com/.

[5] Luzovo. https://www.luvozo.com/.

[6] Bagalà F., Becker C., Cappello A., Chiari L., Aminian K., Hausdorff J.M., Zijlstra W., Klenk J. Evaluation of Accelerometer-Based Fall Detection Algorithms on Real-World Falls.

[7] Bonizzoni E., Puiatti A., Sapienza S., Ros P.M., Demarchi D., Bonato P. UWB Tracking for Home Care Systems with Off-the-Shelf Components. Proceedings of the 2018 IEEE International Symposium on Circuits and Systems (ISCAS).

[8] Yavari M., Nickerson B.G. (2014). Ultra Wideband Wireless Positioning Systems.

[9] Chaaraoui A., Padilla-López J., Ferrandez J., Nieto-Hidalgo M., Flórez-Revuelta F. (2014). A Vision-Based System for Intelligent Monitoring: Human Behaviour Analysis and Privacy by Context. Sensors.

[10] Lu K.L., Chu E.T.H. (2018). An Image-Based Fall Detection System for the Elderly. Appl. Sci..

[11] De Miguel K., Brunete A., Hernando M., Gambao E. (2017). Home Camera-Based Fall Detection System for the Elderly. Sensors.

[12] Williams A., Ganesan D., Hanson A. Aging in Place: Fall Detection and Localization in a Distributed Smart Camera Network. Proceedings of the 15th ACM International Conference on Multimedia; Association for Computing Machinery (MM ’07).

[13] Iliev I., Tabakov S., Dotsinsky I. (2011). Two Steps Approach for Falls Detection in the Elderly. Annu. J. Electron..

[14] Kianoush S., Savazzi S., Vicentini F., Rampa V., Giussani M. (2016). Device-Free RF Human Body Fall Detection and Localization in Industrial Workplaces. IEEE Internet Things J..

[15] Ma L., Liu M., Wang N., Wang L., Yang Y., Wang H. (2020). Room-Level Fall Detection Based on Ultra-Wideband (UWB) Monostatic Radar and Convolutional Long Short-Term Memory (LSTM). Sensors.

[16] Vecchio A., Cola G. Fall detection using ultra-wideband positioning. Proceedings of the 2016 IEEE SENSORS.

[17] Khawaja W., Koohifar F., Guvenc I. UWB radar based beyond wall sensing and tracking for ambient assisted living. Proceedings of the 2017 14th IEEE Annual Consumer Communications Networking Conference (CCNC).

[18] Kolakowski J., Djaja-Josko V., Kolakowski M. (2017). UWB monitoring system for AAL applications. Sensors.

[19] Gharghan S., Mohammed S., Al-Naji A.A. (2018). Accurate Fall Detection and Localization for Elderly People Based on Neural Network and Energy-Efficient Wireless Sensor Network. Energies.

[20] Fung N.M., Wong Sing Ann. J., Tung Y.H., Seng Kheau C., Chekima A. Elderly Fall Detection and Location Tracking System Using Heterogeneous Wireless Networks. Proceedings of the 2019 IEEE 9th Symposium on Computer Applications Industrial Electronics (ISCAIE).

[21] Mynotify. https://www.mynotifi.com/.

[22] Decawave (2015). Source Code Guide, DecaRangeRTLS ARM Source Code, Understanding and Using the DecaRangeRTLS ARM Source Code.

[23] Decawave (2016). TREK1000 Quick Start Guide, Two Way Ranging(TWR) Evaluation Kit.

[24] Weiss A.J., Friedlander B. (1997). Fading effects on antenna arrays in cellular communications. IEEE Trans. Signal Process..

[25] Ghobadi C., Shepherd P.R., Pennock S.R. The study of beyond line-of-sight indoor propagation channels and diversity techniques. Proceedings of the IEE Colloquium on Propagation Characteristics and Related System Techniques for Beyond Line-of-Sight Radio (Ref. No. 1997/390).

[26] Gentner P.K., Hilton G., Beach M.A., Mecklenbräuker C.F. Near and farfield analysis of ultra wideband impulse radio beamforming in the time domain. Proceedings of the 2010 IEEE International Conference on Ultra-Wideband.

[27] Sani A., Alomainy A., Santas J., Yang H. Time domain characterisation of ultra wideband wearable antennas and radio propagation for body-centric wireless networks in healthcare applications. Proceedings of the 2008 5th International Summer School and Symposium on Medical Devices and Biosensors.

[28] Bunin S.G. Data rate in impulse ultra wideband radio networks. Proceedings of the 2010 5th International Confernce on Ultrawideband and Ultrashort Impulse Signals.

[29] Sereewattanapong T., Promwong S. Performance evaluation scheme of ultra wideband impulse radio transmission. Proceedings of the ECTI-CON2010: The 2010 ECTI International Confernce on Electrical Engineering/Electronics, Computer, Telecommunications and Information Technology.

[30] Wang J., Raja A.K., Pang Z. Prototyping and Experimental Comparison of IR-UWB Based High Precision Localization Technologies. Proceedings of the 2015 IEEE 12th Intl Conf on Ubiquitous Intelligence and Computing and 2015 IEEE 12th Intl Conf on Autonomic and Trusted Computing and 2015 IEEE 15th Intl Conf on Scalable Computing and Communications and Its Associated Workshops (UIC-ATC-ScalCom).

[31] Crepaldi M., Ros P.M., Bonanno A., Morello M., Demarchi D. A non-coherent IR-UWB receiver for high sensitivity short distance estimation. Proceedings of the 2014 IEEE International Symposium on Circuits and Systems (ISCAS).

[32] Crepaldi M., Stoppa M., Motto Ros P., Demarchi D. (2015). An Analog-Mode Impulse Radio System for Ultra-Low Power Short-Range Audio Streaming. IEEE Trans. Circuits Syst. I Regul. Pap..

[33] Motto Ros P., Crepaldi M., Bonanno A., Demarchi D. Wireless Multi-channel Quasi-digital Tactile Sensing Glove-Based System. Proceedings of the 2013 Euromicro Conference on Digital System Design.

[34] Shahshahani A., Shahshahani M., Motto Ros P., Bonanno A., Crepaldi M., Martina M., Demarchi D., Masera G. An all-digital spike-based ultra-low-power IR-UWB dynamic average threshold crossing scheme for muscle force wireless transmission. Proceedings of the 2015 Design, Automation Test in Europe Conference Exhibition (DATE).

[35] Damilano A., Motto Ros P., Sanginario A., Chiolerio A., Bocchini S., Roppolo I., Pirri C.F., Carrara S., Demarchi D., Crepaldi M. (2017). A Robust Capacitive Digital Read-Out Circuit for a Scalable Tactile Skin. IEEE Sens. J..

[36] Stoppa M., Ros P.M., Crepaldi M., Chiolerio A., Demarche D. A quasi-digital pressure/touch sensor prototype for orbital targets contact event monitoring. Proceedings of the 2016 IEEE International Symposium on Circuits and Systems (ISCAS).

[37] Motto Ros P., Miccoli B., Sanginario A., Demarchi D. Low-power architecture for integrated CMOS bio-sensing. Proceedings of the 2017 IEEE Biomedical Circuits and Systems Conference (BioCAS).

[38] Sharif Z., Sha’ameri A.Z. The Application of Cross Correlation Technique for Estimating Impulse Response and Frequency Response of Wireless Communication Channel. Proceedings of the 2007 5th Student Conference on Research and Development.

[39] Ozdemir A. (2016). An Analysis on Sensor Locations of the Human Body for Wearable Fall Detection Devices: Principles and Practice. Sensors.

[40] Kononenko I., Šimec E., Robnik-Sikonja M. (1997). Overcoming the Myopia of Inductive Learning Algorithms with RELIEFF. Appl. Intell..

[41] Ozdemir A., Barshan B. (2014). Detecting Falls with Wearable Sensors Using Machine Learning Techniques. Sensors.

[42] Yu K., Guo Y.J., Oppermann I. Modified Taylor Series Expansion Based Positioning Algorithms. Proceedings of the VTC Spring 2008—IEEE Vehicular Technology Conference.

[43] Vicon. https://www.vicon.com/.

[44] Jiménez Ruiz A.R., Seco Granja F. (2017). Comparing Ubisense, BeSpoon, and DecaWave UWB Location Systems: Indoor Performance Analysis. IEEE Trans. Instrum. Meas..

[45] Jiménez A.R., Seco F. Comparing Decawave and Bespoon UWB location systems: Indoor/outdoor performance analysis. Proceedings of the 2016 International Conference on Indoor Positioning and Indoor Navigation (IPIN).

[46] Sang C.L., Adams M., Korthals T., Hörmann T., Hesse M., Rückert U. A Bidirectional Object Tracking and Navigation System using a True-Range Multilateration Method. Proceedings of the 2019 International Conference on Indoor Positioning and Indoor Navigation (IPIN).

