# Supplementary material for: Assessing the Feasibility of Augmenting Fall Detection Systems by Relying on UWB-Based Position Tracking and a Home Robot
Source: Sensors (Basel). 2020 Sep 18;20(18):5361. doi: 10.3390/s20185361 (PMC7570467; doi:10.3390/s20185361)
Supplement: Supplementary file 1 [file sensors-20-05361-s001.zip › sensors-882124 Supplementary Materials xml/supplementary-xml.pdf]

sensors

MDPI

*Article*

# Supplementary Materials

**Maurizio Capra [1,*,†], Stefano Sapienza [2,*,†], Paolo Motto Ros [1], Alessio Serrani [1], Maurizio Martina [1], Alessandro Puiatti [3], Paolo Bonato [2], and Danilo Demarchi [1]**

1 Department of Electrical, Electronics and Telecommunication Engineering, Politecnico di Torino, 10129 Torino TO, Italy; paolo.mottoros@polito.it (P.M.R.); alessio.serrani@polito.it (A.S.); maurizio.martina@polito.it (M.M.); Danilo.demarchi@polito.it (D.D.)

2 Department of Physical Medicine and Rehabilitation, Harvard Medical School, Boston, MA 02129, USA; pbonato@mgh.harvard.edu (P.B.)

3 University of Applied Sciences and Arts of Southern Switzerland Lugano, Swiss 6928; alessandro.puiatti@supsi.ch (A.L.)

* Correspondence: maurizio.capra@polito.it (M.C.); ssapienza@partners.org (S.S.)

† These authors contributed equally to this work.

Received: 13 July 2020; Accepted: 19 August 2020; Published: date

## 1. Supplementary Materials

### 1.1. Ultra-Wide Band (UWB)

UWB is a radio technology based on the transmission of high-frequency pulses (>500 MHz), particularly suitable for ranging and positioning systems [1].

Thanks to the reverse relationship existing between frequency and time domain, the broad bandwidth leads to a short lifetime of the UWB signal and, consequently, to high time resolution that is a crucial feature for positioning [2]. Furthermore, the short-wavelength makes this technology low-power and particularly robust against the multipath in indoor environments [3]. Conventional RF systems transmit information by modulating frequency, power, or phase typical of a sinusoidal wave, while UWB transmits data by generating radio energy at specific time intervals; this makes it suitable for time position and time modulation.

One of the most used UWB transmission methods is the Impulse Radio (IR) [4–7]. In this method, information of the symbol is uttered by the position and/or polarity of the signal. The high time resolution enabled by the IR-UWB approach makes it a good candidate not only for indoor location/tracking systems but also for short distance estimation [8], and, more generally, for all architectures relying on a time-coded or quasi-digital approach [9,10], which in turn allows for the design of very small and simple [11,12] low-power read-out-circuits [13].

The UWB technology is license-free, so anyone can implement a UWB system. However, since UWB covers a wide range of frequencies, regulation must be obeyed that is meant to avoid collisions and interference among different communication protocols. The Federal Communication Commission (FCC) [14] is one of the most important U.S. organizations that issue regulatory requirements. The FCC was the first organization to design rules about UWB in 2002. Many countries, as a result of these regulations, have allocated a frequency spectrum range for UWB use.

### 1.2. Localization Problem

To locate an object in space, relative distances measured concerning fixed points (anchors or nodes) are needed. A certain number of fixed nodes are necessary to find the position of a radio tag. While the object position in 2D can be estimated via the intersection among three circles, as shown in Figure S1, in 3D, the position of the radio tag is derived from spheres built on the basis of at least four nodes. Generally, the positioning process consists of two main steps. First, range values (e.g., distance

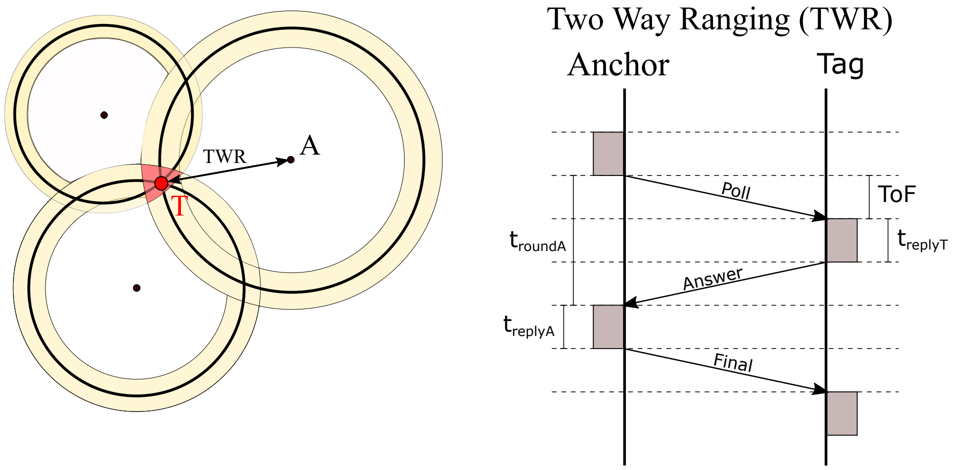


**Figure S1.** Trilateration technique. (left) In the ideal case where the ranges have no uncertainty, and the intersection results in the tag coordinate (red dot). In the real case, the measurements are affected by uncertainty, and the intersection is represented by a surface of points (red area). (right) To minimize the error, the Two Way Ranging (TWR) protocol is applied where the anchor and tag exchange multiple messages.

between anchors and the radio tag) are estimated. Then, the unknown position is found by solving a system of equations. Considering the 2D case (relative to three anchor units), three equations must be solved jointly as follows:

$$d_i = \sqrt{(x_i - x)^2 + (y_i - y)^2} \quad i = 1, 2, 3, \tag{1}$$

where $x$ and $y$ are the tag's coordinates in 2D, $[x_i, y_i]$ is the position of the *i-th* anchor and $d_i$ is the *i-th* range.

One of the most used techniques to estimate the range value is the Time of Flight (ToF). The distance $d$ between two devices (anchor and radio tag) is obtained by measuring the propagation time $\tau$ of a signal that is traveling at a known speed $c$:

$$d = \tau \cdot c \tag{2}$$

The estimated range values are affected by errors. In our case, the ToF is represented by the elapsed time between the time when the signal is transmitted by the radio tag and the time when the anchor unit receives it. The main source of error is hence associated with the synchronization of the electronic boards utilized by different anchor and radio tag units. Even if the clock signal on each board is set at the same frequency, a small clock skews between the transmitter and the receiver can occur. Moreover, since the ToF is on the order of nanoseconds, even small variations can result in significant range variations.

This means that the intersection will not be perfect, or, better yet, the intersection will not result in a specific point, but rather in a delimited area or volume, as shown in Figure S1 (left). The errors that affect the range measurements result in an uncertainty in the radio tag's position that is a function of the accuracy of the ToF estimation.

To improve the accuracy and reduce variability, our units that do not share a global synchronization time axis adopt a process called Two Way Ranging (TWR). Such a technique requires two transceivers (tag and anchor) and a total of three messages to produce a range estimation, as depicted in Figure S1 (right).

Setting $t_{replyB}$ and $t_{replyA}$ to constant time values, when the node A receives the final response message from the node B, it can calculate the ToF by knowing the elapsed time from the poll request:

$$\tau = ToF = \frac{t_{roundA} - t_{replyB}}{2} \tag{3}$$

where $t_{roundA}$ is:

$$t_{roundA} = 2ToF + t_{replyB} \tag{4}$$

the round trip time with respect to node A. By estimating the range (i.e., distance) value using two ToF, it is possible to mitigate the error due to the clock skew. Decawave [15,16] exploits the TWR approach but integrates all anchor units together, so the individual TWRs overlap to save time and be more efficient.

### 1.3. Taylor Series-Based Least Square Algorithm (TSLS)

In this subsection, we will provide some insight into the TSLS algorithm [17] that is applied by the system to correct range values affected by the NLOS condition. Considering an architecture with N fixed anchors, whose coordinates are known ($x_i$, $y_i$, $z_i$ with $i$ = 1, 2, ..., N), and one tag T, whose coordinates need to be computed ($x$, $y$, $z$), the Euclidean distance between $A_i$ and T can be expressed as:

$$d_i(x,y,z) = \sqrt{(x - x_i)^2 + (y - y_i)^2 + (z - z_i)^2} \tag{5}$$

where $i$=1, 2, ..., N. However, the range value $r_i$ measured by the anchor differs from $d_i$ by an error of $\epsilon_i$ that depends on the condition of LOS or NLOS of the transceivers. The state of the anchors can be predicted from the CIR values; consequently, we can define the error for each unit as follows:

$$\epsilon_i = \begin{cases} n_i, & \text{if } i \text{ is in LOS} \\ n_i + L_i & \text{if } i \text{ is in NLOS} \end{cases} \tag{6}$$

where $n_i$ is the measurement error that can be modeled as a zero-mean Gaussian process, while $L_i$ is the distance due to the NLOS condition that can be considered as a Rayleigh distributed random variable.

In general, the coordinates $(\hat{x}, \hat{y}, \hat{z})$ can be expressed as the sum of two components: the true measure plus the error introduced by the obstacle that obstruct the LOS (Equation (7)):

$$x = \hat{x} + \delta_x, y = \hat{y} + \delta_y, z = \hat{z} + \delta_z \tag{7}$$

where $\delta_x$, $\delta_y$ and $\delta_z$ are the NLOS error added. Substituting Equation (7) in Equation (5), expanding using Taylor series around ($\hat{x}$, $\hat{y}$, and $\hat{z}$) and retaining only first two terms, the following equations can be obtained:

$$\begin{cases} a_{i,1}\delta_x + a_{i,2}\delta_y + a_{i,3}\delta_z \simeq r_i - d_i, & \text{if } i \text{ is in LOS} \\ a_{i,1}\delta_x + a_{i,2}\delta_y + a_{i,3}\delta_z \simeq 0, & \text{if } i \text{ is in NLOS} \end{cases} \tag{8}$$

It is possible to observe that in Equation (8) the NLOS corrupted measurement are cancelled out by the estimated distance. This is possible thanks to the definition of $\hat{d}$ and the coefficient $a$ as follows:

$$\hat{d}_i = \sqrt{(\hat{x} - x_i)^2 + (\hat{y} - y_i)^2 + (\hat{z} - z_i)^2}, a_{i,1} = \frac{\hat{x} - x_i}{\hat{d}_i}, a_{i,2} = \frac{\hat{y} - y_i}{\hat{d}_i}, a_{i,3} = \frac{\hat{z} - z_i}{\hat{d}_i} \tag{9}$$

Finally, introducing the matrix form:

$$\mathbf{A} = \begin{bmatrix} a_{1,1} & a_{1,2} & a_{1,3} \\ a_{2,1} & a_{2,2} & a_{2,3} \\ & \cdots\cdots & \\ a_{N,1} & a_{N,2} & a_{N,3} \end{bmatrix} \in R^{Nx3} \tag{10}$$

$$\delta = [\delta_x, \delta_y, \delta_z]^T \in R^{3x1} \tag{11}$$

$$\mathbf{h} = [r_1 - \hat{d}_1 \ldots r_{Nlos} - \hat{d}_{Nlos}]^T \in R^{Nx1} \tag{12}$$

we can apply a Least Square algorithm to the equation:

$$\mathbf{A}\,\delta \simeq \mathbf{h} \tag{13}$$

and compute the residuals $\delta_x$, $\delta_y$ as $\delta_z$ from:

$$\delta \approx (\mathbf{A}^T\mathbf{W}\mathbf{A})^{-1}\mathbf{A}^T\mathbf{W}\mathbf{h} \tag{14}$$

where W is the diagonal matrix of the weights associated with the anchor units. Intuitively, the higher is the weight of a unit, the more reliable is the range value derived from data collected using such unit; in our case, we empirically selected values of 0.1 for the anchor units in NLOS and 1 for the anchor units in LOS. From Equation (14), it is possible to compute $\delta_x$, $\delta_y$, and $\delta_z$ and update the coordinates of the tag:

$$\begin{cases} \hat{x} = \hat{x} + \delta_x \\ \hat{y} = \hat{y} + \delta_y \\ \hat{z} = \hat{z} + \delta_z \end{cases} \tag{15}$$

this process is carried out as an iterative procedure until a stopping condition is reached. Two approaches are possible: setting a threshold on the number of iterations or on the minimum improvement achieved during the iterative process. In our analysis, since all the measures were gathered done off line and computational power was not a concern, we stopped the algorithm after reaching the iteration number 20. However, in a real case scenario, this decision must be taken considering the hardware computational power, to achieve a good trade-off between accuracy and sampling frequency.

#### 1.3.1. Camera-based Motion Capture System

A camera-based motion capture system (Vicon [18] by Oxford Metrics Group, Cambridge UK) was used in the study to monitor the position of the robot during the dynamic tests of the UWB-based position tracking system. This system relies on small reflective markers and infrared cameras that enable tracking the 3D coordinates of the reflective markers with sub-millimeter accuracy.

**Author Contributions:** Investigation, M.C., S.S., A.S.; Resources, P.B. and D.D.; Data curation, S.S.; writing—original draft preparation, M.C.; writing—review and editing, M.C., S.S., P.B. and D.D.; visualization, M.C. and S.S.; supervision, P.M.R., M.M., A.P., P.B. and D.D. All authors have read and agreed to the published version of the manuscript

**Funding:** No external funding supported this research study

**Acknowledgments:** The authors would like to thank Edoardo Bonizzoni for his contribution to testing the WSN system and gathering measures to evaluate the robustness of the alarm transmission.

**Conflicts of Interest:** The authors declare to have no conflicts of interest.

## References


1. Yavari, M.; Nickerson, B.G. *Ultra Wideband Wireless Positioning Systems*; Technical Report; University of New Brunswick: Fredericton, NB, Canada, 2014.
2. Wang, J.; Raja, A.K.; Pang, Z. Prototyping and Experimental Comparison of IR-UWB Based High Precision Localization Technologies. In Proceedings of the 2015 IEEE 12th Intl Conf on Ubiquitous Intelligence and Computing and 2015 IEEE 12th Intl Conf on Autonomic and Trusted Computing and 2015 IEEE 15th Intl Conf on Scalable Computing and Communications and Its Associated Workshops (UIC-ATC-ScalCom), Beijing, China, 10–14 August 2015; pp. 1187–1192.
3. Sani, A.; Alomainy, A.; Santas, J.; Yang, H. Time domain characterisation of ultra wideband wearable antennas and radio propagation for body-centric wireless networks in healthcare applications. In Proceedings of the 2008 5th International Summer School and Symposium on Medical Devices and Biosensors, Hong Kong, China, 1–3 June 2008; pp. 129–132.
4. Bunin, S.G. Data rate in impulse ultra wideband radio networks. In Proceedings of the 2010 5th International Confernce on Ultrawideband and Ultrashort Impulse Signals, Sevastopol, Ukraine, 6–10 September 2010; pp. 141–143.
5. Gentner, P.K.; Hilton, G.; Beach, M.A.; Mecklenbräuker, C.F. Near and farfield analysis of ultra wideband impulse radio beamforming in the time domain. In Proceedings of the 2010 IEEE International Conference on Ultra-Wideband, Nanjing, China, 20–23 September 2010; Volume 2, pp. 1–4.
6. Sereewattanapong, T.; Promwong, S. Performance evaluation scheme of ultra wideband impulse radio transmission. In Proceedings of the ECTI-CON2010: The 2010 ECTI International Confernce on Electrical Engineering/Electronics, Computer, Telecommunications and Information Technology, Chiang Mai, Thailand, 19–21 May 2010; pp. 405–409.
7. Crepaldi, M.; Stoppa, M.; Motto Ros, P.; Demarchi, D. An Analog-Mode Impulse Radio System for Ultra-Low Power Short-Range Audio Streaming. *IEEE Trans. Circuits Syst. I Regul. Pap.* **2015**, *62*, 2886–2897.
8. Crepaldi, M.; Ros, P.M.; Bonanno, A.; Morello, M.; Demarchi, D. A non-coherent IR-UWB receiver for high sensitivity short distance estimation. In Proceedings of the 2014 IEEE International Symposium on Circuits and Systems (ISCAS), Melbourne VIC, Australia, 1–5 June 2014; pp. 1905–1908.
9. Motto Ros, P.; Crepaldi, M.; Bonanno, A.; Demarchi, D. Wireless Multi-channel Quasi-digital Tactile Sensing Glove-Based System. In Proceedings of the 2013 Euromicro Conference on Digital System Design, Los Alamitos, CA, USA, 4–6 September 2013; pp. 673–680.
10. Shahshahani, A.; Shahshahani, M.; Motto Ros, P.; Bonanno, A.; Crepaldi, M.; Martina, M.; Demarchi, D.; Masera, G. An all-digital spike-based ultra-low-power IR-UWB dynamic average threshold crossing scheme for muscle force wireless transmission. In Proceedings of the 2015 Design, Automation Test in Europe Conference Exhibition (DATE), Grenoble, France, 9–13 March 2015; pp. 1479–1484.
11. Damilano, A.; Motto Ros, P.; Sanginario, A.; Chiolerio, A.; Bocchini, S.; Roppolo, I.; Pirri, C.F.; Carrara, S.; Demarchi, D.; Crepaldi, M. A Robust Capacitive Digital Read-Out Circuit for a Scalable Tactile Skin. *IEEE Sens. J.* **2017**, *17*, 2682–2695.
12. Stoppa, M.; Ros, P.M.; Crepaldi, M.; Chiolerio, A.; Demarche, D. A quasi-digital pressure/touch sensor prototype for orbital targets contact event monitoring. In Proceedings of the 2016 IEEE International Symposium on Circuits and Systems (ISCAS), Montreal, QC, Canada, 22–25 May 2016; pp. 2843–2846.
13. Motto Ros, P.; Miccoli, B.; Sanginario, A.; Demarchi, D. Low-power architecture for integrated CMOS bio-sensing. In Proceedings of the 2017 IEEE Biomedical Circuits and Systems Conference (BioCAS), Turin, Italy, 19–21 October 2017; pp. 1–4.
14. Federal Communications Commission. *Bell Labs Technical Journal*; Federal Communications Commission: Washington, DC, USA, 2004, Volume 9.
15. Decawave. *TREK1000 Quick Start Guide, Two Way Ranging(TWR) Evaluation Kit*, 1.3 edition; Decawave: Dublin, Ireland, 2016.
16. Decawave. *Source Code Guide, DecaRangeRTLS ARM Source Code, Understanding and Using the DecaRangeRTLS ARM Source Code*, 2.2 edition; Decawave: Dublin, Ireland, 2015.
17. Yu, K.; Guo, Y.J.; Oppermann, I. Modified Taylor Series Expansion Based Positioning Algorithms. In Proceedings of the VTC Spring 2008 - IEEE Vehicular Technology Conference, Singapore, 11–14 May 2008; pp. 2656–2660.

18. Vicon. Available online: https://www.vicon.com/ (accessed on 7 August 2020).

© 2020 by the authors. Licensee MDPI, Basel, Switzerland. This article is an open access article distributed under the terms and conditions of the Creative Commons Attribution (CC BY) license (http://creativecommons.org/licenses/by/4.0/).
