# Supplementary figures and images for: Assessing the Feasibility of Augmenting Fall Detection Systems by Relying on UWB-Based Position Tracking and a Home Robot
Source: Sensors (Basel). 2020 Sep 18;20(18):5361. doi: 10.3390/s20185361 (PMC7570467; doi:10.3390/s20185361)

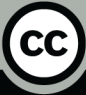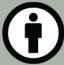

BY

Supplement: Supplementary file 1 [file sensors-20-05361-s001.zip › sensors-882124 Supplementary Materials xml/Definitions/logo-ccby-eps-converted-to.pdf]

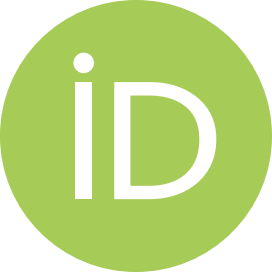

Supplement: Supplementary file 1 [file sensors-20-05361-s001.zip › sensors-882124 Supplementary Materials xml/Definitions/logo-orcid-eps-converted-to.pdf]

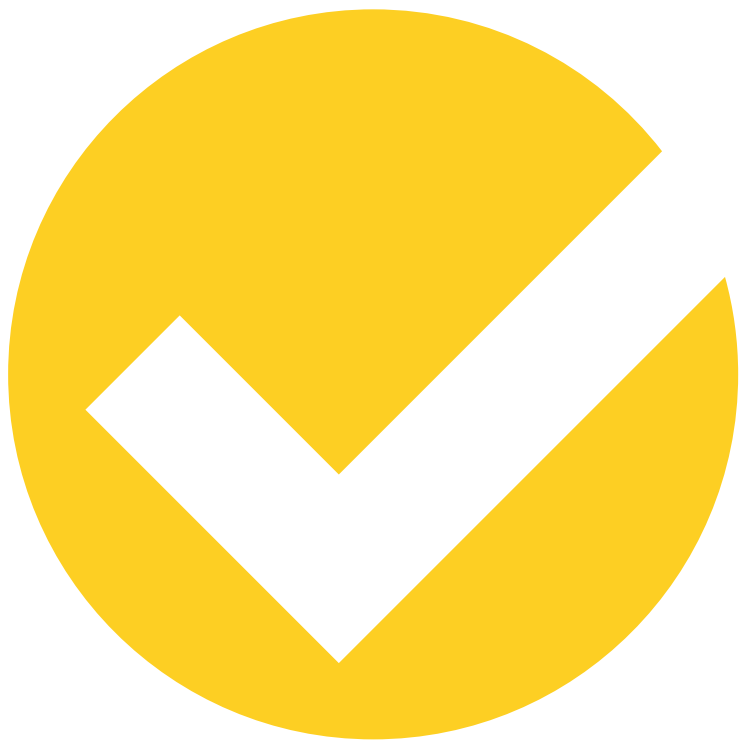

check for  
updates

Supplement: Supplementary file 1 [file sensors-20-05361-s001.zip › sensors-882124 Supplementary Materials xml/Definitions/logo-updates.pdf]

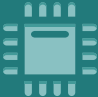

*sensors*

Supplement: Supplementary file 1 [file sensors-20-05361-s001.zip › sensors-882124 Supplementary Materials xml/Definitions/sensors-logo-eps-converted-to.pdf]

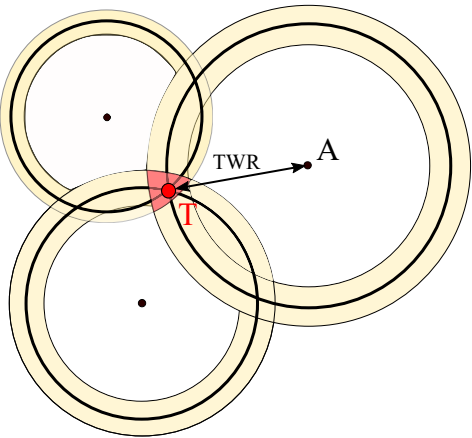

## Two Way Ranging (TWR)

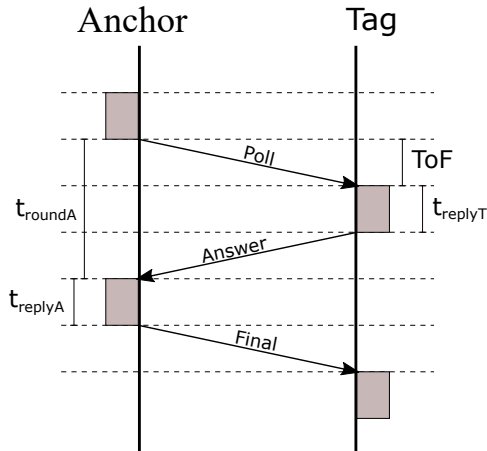

Supplement: Supplementary file 1 [file sensors-20-05361-s001.zip › sensors-882124 Supplementary Materials xml/imm/positioning2.pdf]
